# Annexins—Coordinators of Cholesterol Homeostasis in Endocytic Pathways

**DOI:** 10.3390/ijms19051444

**Published:** 2018-05-12

**Authors:** Carles Rentero, Patricia Blanco-Muñoz, Elsa Meneses-Salas, Thomas Grewal, Carlos Enrich

**Affiliations:** 1 Departament de Biomedicina, Unitat de Biologia Cel·lular, Facultat de Medicina i Ciències de la Salut, Universitat de Barcelona. 08036 Barcelona. Spain; patrii__4@live.com (P.B.-M.); elsameneses.s@gmail.com (E.M.-S.); enrich@ub.edu (C.E.); 2 School of Pharmacy, Faculty of Medicine and Health, University of Sydney, Sydney, NSW 2006, Australia; thomas.grewal@sydney.edu.au; 3 Centre de Recerca Biomèdica CELLEX, Institut d’Investigacions Biomèdiques August Pi i Sunyer (IDIBAPS), 08036 Barcelona, Spain

**Keywords:** Annexins, cholesterol, Ca^2+^, signalling, lysosomes, late endosomes

## Abstract

The spatiotemporal regulation of calcium (Ca^2+^) storage in late endosomes (LE) and lysosomes (Lys) is increasingly recognized to influence a variety of membrane trafficking events, including endocytosis, exocytosis, and autophagy. Alterations in Ca^2+^ homeostasis within the LE/Lys compartment are implicated in human diseases, ranging from lysosomal storage diseases (LSDs) to neurodegeneration and cancer, and they correlate with changes in the membrane binding behaviour of Ca^2+^-binding proteins. This also includes Annexins (AnxA), which is a family of Ca^2+^-binding proteins participating in membrane traffic and tethering, microdomain organization, cytoskeleton interactions, Ca^2+^ signalling, and LE/Lys positioning. Although our knowledge regarding the way Annexins contribute to LE/Lys functions is still incomplete, recruitment of Annexins to LE/Lys is greatly influenced by the availability of Annexin bindings sites, including acidic phospholipids, such as phosphatidylserine (PS) and phosphatidic acid (PA), cholesterol, and phosphatidylinositol (4,5)-bisphosphate (PIP2). Moreover, the cytosolic portion of LE/Lys membrane proteins may also, directly or indirectly, determine the recruitment of Annexins to LE. Strikingly, within LE/Lys, AnxA1, A2, A6, and A8 differentially contribute to cholesterol transport along the endocytic route, in particular, cholesterol transfer between LE and other compartments, positioning Annexins at the centre of major pathways mediating cellular cholesterol homeostasis. Underlying mechanisms include the formation of membrane contact sites (MCS) and intraluminal vesicles (ILV), as well as the modulation of LE-cholesterol transporter activity. In this review, we will summarize the current understanding how Annexins contribute to influence LE/Lys membrane transport and associated functions.

## 1. Introduction

Annexins are a large protein family that is expressed in vertebrates, invertebrates, plants, fungi, and protists, which bind to biological membranes in a Ca^2+^-dependent manner [1]. In humans, the 12 different Annexin proteins (AnxA1–A11, A13) [2] all consist of a highly conserved core domain that comprises four structural repeats, each 70–75 amino acid residues in length, and containing type II Ca^2+^ binding sites. In addition, providing specificity, each Annexin is endowed with a unique N-terminal domain of different length. Most likely due to gene duplication, AnxA6 is the only family member consisting of two copies of the four-repeat core domains that are connected by a flexible linker region [3,4,5,6,7].

When considering the miscellany of Ca^2+^-related events at cellular membranes, Annexins contribute to a variety of intracellular membrane trafficking steps, but also membrane that are associated signalling, altogether influencing proliferation, differentiation, and inflammation [2,8,9,10]. Over the last few decades, the Ca^2+^-dependent membrane association of Annexins has been investigated intensively in endo- and exocytosis, the spatial organization of plasma membrane lipids, in particular, during membrane repair, as well as in the linkage of membranes to the actin cytoskeleton [1,11,12,13,14,15]. In addition to these properties that are driven by Annexin-lipid interactions, Annexins associate with a plethora of other proteins, including Ca^2+^-effectors, and can form Ca^2+^-permeable ion channels in artificial membranes [16,17,18,19,20,21,22,23], providing connections to Ca^2+^ homeostasis and Ca^2+^-driven signal transduction [1,3,24].

Here, we will review the role of Annexins that are localized in the late endosomes (LE)/lysosomes (Lys) compartment as Ca^2+^ effectors for the regulation of cholesterol transport, signalling, and homeostasis in vesicular and non-vesicular trafficking pathways.

## 2. Late Endocytic Compartments

The endocytic compartment is a functional continuity of the plasma membrane, connecting the extracellular environment with the perinuclear region, where LE fuse with Lys, the endpoint of the constitutive endocytic pathway. The vast complexity of the endocytic compartment is reflected by a sophisticated molecular repertoire of proteins and lipids that are in charge of organizing the characteristic membrane morphology of each compartment, and functioning of membrane and cargo transport between these different endocytic entities. Moreover, new insights in the transport between cellular organelles strongly implicate additional non-vesicular pathways to fuel the exchange of membrane lipids, such as cholesterol and ions, including Ca^2+^ (see Section 6) [25,26].

In the following, we will focus on the late endocytic compartment, which includes LE, multivesicular bodies (MVB), pre-lysosomes, endolysosomes, autophagosomes, amphisomes, Lys, and autolysosomes. Most of those late endocytic structures continuously undergo maturation processes before fusion with Lys, which is considered to be crucial to acquire identity and differential functionality [27].

The concept of maturation from early endosomes (EE) to LE and beyond encompasses an array of diverse cellular events that eventually generate new morphologically and functionally different compartments. This heterogeneity among LE structures is reflected in the diversity of ultrastructural details that were identified by electron microscopy [28]. Critical changes along the maturation from EE to LE/Lys include: (i) the formation of intraluminal vesicles (ILV), (ii) the acidification and changes in luminal Ca^2+^ levels, (iii) the conversion of phosphatidylinositol (PI), contributing to alterations in size and morphology, (iv) changes in fusion specificity and motility, and (v) the gain of degradative potential by the accumulation of hydrolases [29,30]. In contrast, and occurring simultaneously, there is a major loss of recycling capacity, most probably as a consequence of decreased membrane extensions and the eventual acquisition of a vacuolar shape.

Closely interconnected with LE maturation and functioning, LE movement is crucial for fusion with other endocytic or autophagic structures and determines the position in the cell, which is now well recognized as highly relevant for cell physiology (see Section 8). Microtubules, but also actin microfilaments, facilitate LE movement. A myriad of microtubule-associated, actin binding, and cytosolic proteins, together with Ca^2+^ signals, fine-tune the assembly/disassembly of protein complexes that associate LE with tubules and fuel the motor proteins to control the directionality of LE vesicle movement [31,32].

**Figure 1:**
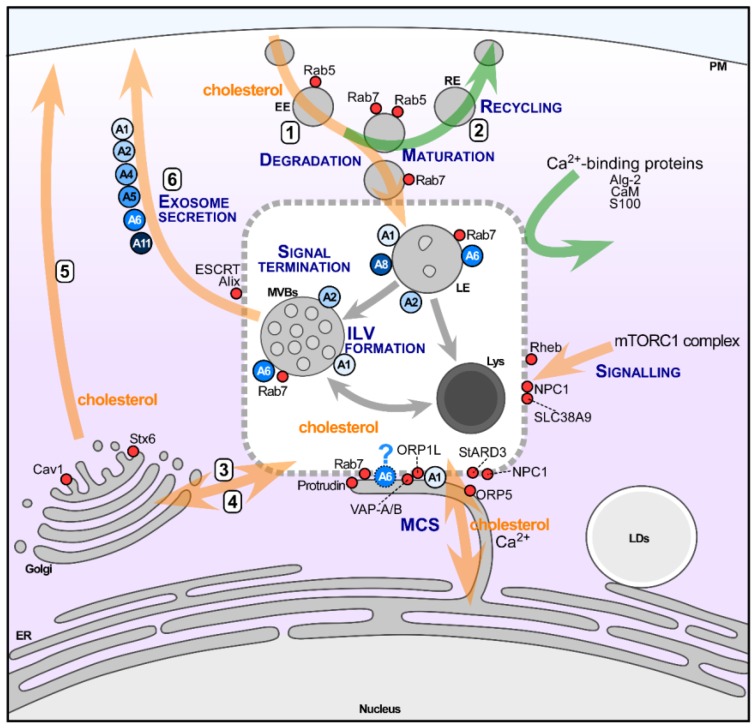
Schematic overview of Annexins at the crossroad of late endocytic pathways. Late endocytic structures (LE), MVBs containing ILV and Lys with associated Annexins are depicted in the centre of the diagram. The LE compartment dynamically and functionally interacts with several inbound and outbound routes; (1) maturation of early endosomes (EE); (2) the recycling pathway to the plasma membrane; (3) the transport route for the biogenesis of lysosomes from Golgi or (4) the retrograde trafficking to the Golgi membranes. Rab proteins (i.e., Rab5, 7) are critical for these pathways are also shown. In addition, a subset of cytosolic proteins, such as Ca^2+^ binding proteins (i.e., calmodulin, CaM; S100 family; apoptosis-linked gene 2, Alg-2) and signalling proteins (i.e., mammalian target of rapamycin complex 1, mTORC1), interact with proteins (lipids) at the membrane of LE/Lys, contributing to the regulation of ion channels, pumps, enzymes or signalling complexes. The close connection with ER membranes enables membrane contact sites (MCS) to establish metabolic functional platforms for the exchange of lipids (cholesterol) and ions (Ca^2+^). Specific proteins, “tethers”, such as AnxA1 and possibly AnxA6, or “exchangers”, like StARD3, ORP1L, or ORP5 at the LE/Lys membrane, are attached via two phenylalanines acid track (FFAT) motifs with vesicle-associated membrane protein-associated proteins A/B (VAP-A/B) or protrudin at endoplasmic reticulum (ER) membranes. Hence, complex interplay of vesicular transport with non-vesicular transport through MCS guarantee Ca^2+^ and cholesterol homeostasis and the positioning of LE/Lys constituents. Although Annexins are commonly considered cytosolic proteins, they have been identified inside as well as outside LE structures. Ca^2+^, acidic phospholipids and cholesterol regulate the recruitment and binding of a subset of Annexins to the LE surface; Finally, (5) the secretory pathway for constitutive exocytosis and (6) a regulated transport for the secretion of exosomes, involving ESCRT and Alix from MVBs, are shown. Orange arrows indicated those pathways modulated by cholesterol. Green arrows indicated recycling pathways and cytoplasmic proteins interacting with late endocytic compartment. Grey arrows indicate maturation of the late endocytic structures. Caveolin-1, Cav1; Syntaxin 6, Stx6; StARD3, StAR-related lipid transfer domain protein 3; ORP1L, oxysterol-binding protein-related protein 1, ESCRT, endosomal-sorting complexes required for transport, Alix, Alg-2 interacting protein X. See text for further details.

Several protein families represent well-established LE markers, having fundamental tasks for the proper functioning of this compartment. This includes Rab and SNARE proteins, together with other tethering protein families, with many excellent reviews that are covering the regulatory role of these proteins in much detail [33,34]. However, far less well-characterized are the plethora of cytosolic proteins, including peripheral membrane components, but also signalling, Ca^2+^ (for example Annexins or calmodulin), or actin binding proteins that support, regulate, and define these late endocytic structures (Figure 1). 

## 3. Annexins in Late Endocytic Compartments

Accumulating evidence underscores the tight association of Annexins with the functioning of the endocytic compartment. AnxA1 was first identified to be necessary for EE fusion in a Ca^2+^-dependent manner [15], followed by evidence for its involvement in the inward vesiculation of MVBs [35], and more recently, MCS formation between the endoplasmic reticulum (ER) and LE/MVBs [36] (see Section 6). Likewise, AnxA2, A5, and A6 can bring together EE [37], autophagosomes/lysosomes [38], and LE/Lys [39], respectively. Annexins are now commonly believed to drive these fusion events via their ability to function as organizers of membrane domains, in order to target their interaction partners to specific membrane microdomains and enable the formation of compartment-specific complexes and activities [1,2,9,13,39,40]. In addition, several Annexins, including AnxA2 and AnxA6, also contribute to the segregation of membrane lipids and the re-arrangement of membrane-cytoskeleton interactions to promote membrane curvature, a prerequisite for the budding of vesicles [41,42,43,44]. The ability of AnxA1 and AnxA8 to coordinate the contacts between membrane lipids and the actin cytoskeleton [45,46,47] may further contribute to vesicle budding.

The findings that are listed above implicate Annexins to participate in the maturation of the endocytic pathway. LE structures have a high negative cytosolic surface charge [48,49] and they are enriched with phospholipids, such as PS, PA, and PI [50,51]. The negative charge suggests that LE/Lys can serve as a docking site for proteins with PS-binding C2-domains, which include signalling and fusogenic effectors, but also Annexins [1,2,3]. This Ca^2+^-dependent binding to phospholipids is a fundamental property of Annexins, and it provides the basis for reversible Annexin membrane binding via fluctuation in localized Ca^2+^ concentrations. Thus, AnxA1, A2, A5, A6 and A8 can all be found in LE/Lys, yet pools/subpopulations of these Annexins can also interact with biological membranes in the absence of Ca^2+^ [52,53,54,55]. This is exemplified by AnxA2-dependent endosome maturation, which represents an example for cholesterol-driven LE membrane binding. This well-studied contribution of AnxA2 in the budding of vesicles from EE to form LE [56] probably occurs in a p11/S100A10-dependent manner [57], and it requires the phosphorylation of the AnxA2 N-terminal region [58]. In this scenario, AnxA2, together with the Spire Type Actin Nucleation Factor 1, induces actin patch formation in EE, which ultimately drives membrane remodelling and budding [58]. Similarly, AnxA1 phosphorylation [36,52] and the interaction with S100A11 [36,59] contribute to MVB vesiculation in a cholesterol-sensitive manner. Likewise, and as described in more detail below, the association of AnxA6 with LE is sensitive to cholesterol levels [3,60,61]. Taken together, this clearly highlights membrane binding properties, protein interactions with other Ca^2+^ effectors, and signalling events as drivers for Annexin-dependent endosomal functions (Figure 1).

Further adding to the complexity that determines the LE membrane association of Annexins, Ca^2+^ can promote vesicle fusion by inducing the local segregation/re-arrangements of lipids, such as PA or cholesterol [40,62,63,64,65]. Given that LE/Lys function as acidic Ca^2+^-stores, with several integral Ca^2+^ transporters shuttling Ca^2+^ across the LE/Lys membrane, this would allow for Ca^2+^ fluctuations to affect phospholipid binding affinity of Annexins, but also the localized availability of cholesterol for LE/Lys membrane association. In fact, for membrane fusion, local Ca^2+^ is crucial and acidic intracellular Ca^2+^ stores are well integrated in this process. One excellent example for Annexins linking Ca^2+^-homeostasis with lysosomal function is the interactome of two pore segment channel 1/2 (TPC1/2) proteins, Ca^2+^ channels that contain several members of the Annexin family [66].

Finally, AnxA8, which is similar to AnxA2 in other locations, binds to PIP2 and actin in a Ca^2+^-dependent manner in LE [46,47]. As other LE-associated Annexins also interact with actin [1,13], one can envisage that actin-related recruitment of Annexins to the LE/Lys compartment is not restricted to AnxA8. Hence, Annexins may contribute to membrane fusion via Ca^2+^-dependent and -independent membrane and protein interactions.

One defining feature of Annexins is their capacity to bind to negatively charged lipids of cellular membranes, in particular, PS, PA, and PI, in a Ca^2+^-dependent manner [1,3]. However, the fact that Annexins have been located in a variety of organelles in living cells, irrespective of Ca^2+^ levels and negatively charged phospholipids [1,3], pointed at additional mechanisms to target and to modulate the binding of Annexins to membranes. This coincides with the abovementioned cholesterol-dependent membrane binding of several Annexins [62], which becomes highly relevant in the context of LE, representing the main sorting compartment for low density lipoprotein (LDL)-derived cholesterol [67,68]. In fact, pathological settings, such as LSDs, neurological disorders, and many others, are characterized by cholesterol accumulation in the LE compartment, which drives the LE membrane association of several Annexins, including the most abundant Annexins in LE, AnxA1, A2, A5, and A6 [36,38,69,70]. This may point at a direct interaction of Annexins with cholesterol [71], an observation that is further supported by the identification of Annexins as cholesterol-binding proteins in a proteome-wide mapping approach in living cells [72]. Likewise, the fact that those Annexins are also highly enriched in cholesterol-rich lipid rafts [73] further substantiates cholesterol to promote Annexins association with LE membranes. In favour of this concept, AnxA2 and AnxA6 bind to membranes in a cholesterol-sensitive manner in vitro [61,63] and in cells [60,62]. Also, a fraction of AnxA2 requires cholesterol, but not Ca^2+^, to bind to chromaffin granules [63], mainly regulated by the Annexin core domain [62]. Similarly, AnxA6 associates with high affinity to lipid monolayers with increased amounts of cholesterol at acidic pH. This cholesterol-dependent membrane interaction requires the tryptophan 343 residue within the linker region between the two core domains of AnxA6 [61]. Most strikingly, cell-based studies revealed calcium-insensitive, but cholesterol-dependent, binding properties of a pool of AnxA6 proteins to LE membranes [60]. In addition, the structural flexibility of AnxA6 between its two core domains provides opportunity to bind two membranes simultaneously [1], making AnxA6 a suitable candidate to tether organelles and to participate in membrane fusion/docking during endocytic transport, but also MCS formation.

## 4. Annexins and Cholesterol Homeostasis

Cholesterol can be synthesized by cells, but the majority of cellular cholesterol is supplied by LDL endocytosis. After internalization, esterified LDL-cholesterol reaches LE/Lys, where it is hydrolysed to be delivered to other sites via NPC1/2 proteins [67,68]. Up to 30% of LDL-cholesterol moves to the ER, to regulate feedback control of cholesterol biosynthesis [68,74]. From the ER, cholesterol can then be delivered to other organelles, such as the plasma membrane or mitochondria. Alternatively, excess cholesterol can be esterified by acyl-coenzyme A:cholesterol acyltransferase for storage in lipid droplets [75]. Most relevant to this review, increasing evidence suggests that MCS between endosomes and the ER also control the cellular distribution of cholesterol [76,77].

The cholesterol-binding properties of Annexins that are described above probably have far reaching consequences not only for the membrane order of certain membranes, possibly contributing to stabilize or to create specific microdomains, but also for cellular cholesterol homeostasis. For instance, the ability of AnxA1 to establish membrane contacts between the ER and MVBs not only ensures the downregulation of EGFR, but also is accompanied by the transfer of cholesterol from the ER to MVBs. This is an unusual cholesterol transport route, as cells commonly obtain cholesterol through LDL endocytosis and LDL-derived cholesterol in LE is normally transferred to the ER to downregulate *de novo* cholesterol synthesis [67,68]. However, when LDL-derived cholesterol levels in MVBs are low, this reverse ER to LE route of sterol traffic seems to ensure the presence of cholesterol as an essential factor for inward vesiculation and ILV formation [36].

In the case of AnxA2, increased association of this Annexin with cholesterol-rich LE was observed [56]. This correlated with an accumulation of EGF ligands in EE upon AnxA2 depletion, suggesting a cholesterol-dependent role for AnxA2 in the delivery of transport intermediates to LE [56]. However, these findings might be restricted to certain cell types [60], and other studies identified AnxA2 knockdown to not interfere with the degradative pathway, but the recycling of the transferrin receptor [78].

We unravelled several significant cellular alterations due to AnxA6 binding to LE membranes, all being relevant to the distribution of cholesterol and functionality of other cellular compartments [3]. AnxA6 overexpression led to increased amounts of AnxA6 in the LE compartment, which was associated with (i) accumulation of cholesterol in LE/Lys, while cholesterol levels at the plasma membrane, Golgi, and recycling endosomes were reduced [69,79,80]. (ii) This Niemann-Pick type C1 (NPC1) mutant-like phenotype triggered the sequestration of caveolin-1 in the Golgi, leading to reduced numbers of cholesterol-rich caveolae at the cell surface [79]. (iii) AnxA6-induced changes in cellular cholesterol distribution also interfered with the trafficking of cholesterol-dependent SNARE proteins (e.g., syntaxin 4, syntaxin 6, SNAP23) and integrins, compromising their critical functions in cell adhesion and migration [69,80,81]. The latter findings might be indirectly linked with a potential role for SNARE proteins in cellular cholesterol trafficking. First, SNAREs represent cholesterol-sensitive components of membrane fusion and vesicular transport, and their function and localization is influenced by the cholesterol levels in the endocytic compartment [67,82]. Second, several SNAREs directly interact with cholesterol [67,72,83]. These observations might extend to other Annexins, as AnxA2 translocates to SNARE proteins during exocytosis [84]. Hence, the influence of Annexins on SNARE-mediated membrane fusion and docking may not only deliver cargo to specific destinations, but also serve to transport cholesterol between cellular compartments.

The molecular means how the recruitment of AnxA6 to LE alters LE-cholesterol transport is not fully understood, but it probably involves specific protein-protein interactions that enable AnxA6 to block LE-cholesterol egress (see Section 4 and Section 6). However, AnxA6 membrane binding to trigger the remodelling of cholesterol-rich microdomains, as shown to occur at the plasma membrane, should also be considered [61,73,85]. These lipid-binding features of AnxA6 may cause similar domain changes in the LE compartment, thereby modulating the spatial distribution of cholesterol, and consequently other lipids, in LE membranes, creating specific microenvironments, such as membrane rafts [61,85,86]. These highly ordered and cholesterol-rich domains may influence cholesterol transporter activity, including NPC1, provide the platform for proteins to establish MCS (see Section 6), or contribute to control the formation of signal transduction platforms that are linked to Ca^2+^ homeostasis, LE-cholesterol egress, LE maturation, or other LE/Lys functions, such as growth factor receptor signal termination.

Finally, in addition to AnxA6, AnxA8 also controls LE-cholesterol homeostasis [87]. Yet, strikingly opposite to the requirement of AnxA6 upregulation for LE-cholesterol accumulation, only AnxA8 depletion caused the blockage of LE-cholesterol egress, suggesting a possible counter-balance of these two Annexins in this context. Taken together, several Annexins impact on cholesterol transport at various steps along the endocytic pathway, and their up- and downregulation is differentially contributing to the intricate network of feedback mechanisms that are associated with cellular cholesterol homeostasis.

## 5. Annexins and Signalling in Late Endocytic Compartments

The endocytic pathway is characterized by compartment-specific microdomains that (1) ensure compartment identity and directional trafficking, and (2) enable diversity of signal transduction either from multiple or localized endocytic entities. 

The maturation of endosomes along endocytic pathways is orchestrated by compartment-specific Rab proteins. Different members of these small monomeric GTPases are found in specific endosomal subpopulations regulating endosomal position, movement, and fate [33,34]. The subcellular distribution of Rab proteins within the endocytic membrane network is closely connected to signalling platforms that control the location and the activation status of receptors along the degradative pathway, but also signalling events along the recycling of receptors and ligands [33,34]. In most cases, signalling cascades are triggered by receptor activation at the cell surface. The classical example is the epidermal growth factor receptor (EGFR)-induced activation of the Ras/mitogen-activated protein kinase (MAPK) pathway, which is initiated at the plasma membrane, and it continues to signal throughout the endocytic pathway as all components, including EGFR, Ras, Raf-1, and MAPK traffic through EE and LE compartments [88].

On the other hand, very localized and endosome-specific signalling complexes exist, such as the mammalian target of rapamycin complex 1 (mTORC1). This protein complex drives energy metabolism in cells and it is specifically recruited to LE/Lys upon activation [89].

As outlined below, several Annexins appear to modulate various aspects of endosome trafficking and signalling along the degradation pathway in LE, MVBs, autophagosomes, and Lys.

### 5.1. Regulation of Endosomal Fate: Rab Proteins

Major advances in the understanding of endocytic membrane transport have come from the identification of Rab GTPases as markers for different endosomal compartments [33,34]. The large Rab GTPase family comprises approximately 70 members in humans, with the majority of these Rab proteins (~75%) acting alongside endocytic trafficking routes. Each Rab protein is localized in membrane microdomains of a specific endocytic compartment to organize a collection of specific effectors that enable endosome maturation, receptor trafficking, and signal transduction. For instance, the maturation of endocytic vesicles down the degradative route is ensured by the progressive substitution of particular Rab GTPases by others decorating the endosomal membrane.

The coordination of these so-called Rab cascades is complex, and is based on Rab GTPases that are acting as molecular switches that alternate between active GTP-bound and inactive GDP-bound states. This is facilitated by their specific, cognate guanine nucleotide exchange factors (GEFs) and GTPase-activating proteins (GAPs), which regulate RabGTP/GDP levels of a specific Rab protein in response to environmental changes, ultimately policing other Rabs acting up- and/or downstream. This multifactorial machinery thereby establishes the identity of organelles, determines compartmentalization of early, late, lysosomal, and recycling routes, allows for vesicle budding and fusion, and integrates signalling cascades.

While Rab5 critically determines EE functionality, the LE/MVB/Lys compartment is defined by Rab7, Rab9, and Rab24, which control lysosome biogenesis, autophagosomal maturation, and vesicle transport through the interaction with multiple effector proteins [34,90]. During the maturation from EE to LE, the EE marker Rab5 is progressively substituted by Rab7. In brief, the current models favour Rab5 and PIP2 to recruit the protein complex MON1A/B-CCZ1, which reduces Rab5 activity. Rab5 is then released from the membrane, enabling MON1A/B-CCZ1 to recruit and activate Rab7 [29]. Alternatively, the budding and fission of Rab7 domains present on Rab5-positive endosomes may also contribute to EE maturation [91]. Progressing from LE to Lys entails further regulatory steps, requiring other Rab proteins, in particular, Rab9, which mediates the sorting of lysosomal enzymes and lipids from the trans-Golgi-network to Lys and autophagosomes [92,93].

Besides PIP2 and PS contributing to regulate the association and function of Rab proteins in LE/Lys, cholesterol has also been identified to modulate Rab behaviour in LE/Lys. Hence, the ability of AnxA1, A2, A6, and A8 to influence cholesterol transport within endosomal compartments (see Section 4) is likely to affect Rab-GTPase activities in EE and LE/Lys. How AnxA1-mediated cholesterol transport from the ER to MVB [36] or AnxA2-dependent formation of cholesterol-rich platforms in EE for the onset of degradation [56] could affect Rab functionality is unclear, but several studies addressing Rab activity after LE-cholesterol accumulation provides some insight into the possible alterations of Rab-GTP/GDP cycles in LE/Lys upon AnxA6 overexpression or AnxA8 depletion. For instance, in NPC1 mutant cells, LE-cholesterol accumulation sequesters Rab9 and disrupts LE function, as judged by the missorting of mannose 6-phosphate receptor to Lys for degradation. At the molecular level, this involves impaired Rab9 protein turnover, as increased cholesterol in NPC1 mutant membranes interfered with the extraction of inactive Rab9 protein via GDP dissociation inhibition proteins (GDIs) [94]. Likewise, LE-cholesterol accumulation also impairs the GTP/GDP cycle of Rab7a [95], thus reducing LE motility. In these earlier studies, increased LE-cholesterol was proposed to interfere with GDI-dependent removal of inactive Rab7 from LE membranes [95]. Based on these studies, up- or downregulation of AnxA6 and AnxA8, respectively, could act similarly to cause detrimental effects on the Rab9 and the Rab7 GTP/GDP cycle. However, the results from our laboratories may provide an alternative explanation for the latter observation. Similar to the scaffolding function of AnxA6 at the plasma membrane, where AnxA6 reduces Ras-GTP levels via the recruitment of a Ras-GAP family member, p120GAP, we recently identified AnxA6 upregulation to reduce Rab7-GTP levels in NPC1 mutant cells, possibly via the recruitment of a Rab7-GAP to cholesterol-rich LE. Taken together, and revisiting models that are proposed in earlier studies [96,97,98,99,100], these findings implicate regulatory roles of several Annexins, through modulation of cholesterol transport or direct protein interactions, for Rab proteins in LE/Lys, key players in the endpoint of the endocytic pathway.

### 5.2. The Coordination of EGFR Signalling and Trafficking

The regulation of EGFR activity is probably the best-studied example for the tight coordination of signalling and trafficking along the endocytic pathway. Upon ligand binding at the cell surface, EGFR dimerization and activation triggers the binding of adaptors, which activate multiple signalling cascades that regulate cell proliferation, migration, and many other cellular activities [101,102]. To avoid constitutive signalling, ligand binding simultaneously stimulates rapid EGFR internalization, targeting active EGFR and its downstream effectors through the endocytic system for degradation in the LE/Lys compartment. While EGFR signalling was initially considered to exclusively occur at the plasma membrane, it is now well documented that EGFR signal output is overseen by compartmentalization, providing opportunity for signal specificity, and that EGFR trafficking down the degradative route relies on signalling outcomes. Indeed, numerous studies have provided evidence that within EE, Rab5 and its effectors are critical for the proper targeting of activated EGFR to the LE/Lys compartment, but also for EGFR signalling magnitude in the EE compartment [103]. Likewise, the Rab7 interactome ensures EGFR downregulation in lysosomes, which is critical for EGFR signal termination [30]. On the other hand, EGFR phosphorylation improves the interaction with assembly polypeptide 2 complex for endocytosis [101,104,105], and phosphorylation of EGFR substrate 15 is decisive for EGFR internalisation [106].

Interestingly, cells are able to modify the magnitude of EGFR activation via different internalization routes. Low amounts of EGF trigger clathrin-mediated endocytosis that target EGFR to Rab5-positive EE, which are then destined to the perinuclear region. The simultaneous increase of EGFR phosphatase activity in this compartment ultimately promotes EGFR recycling to the plasma membrane. In contrast, high amounts of EGF induce substantial EGFR phosphorylation, but also EGFR ubiquitination, which favours clathrin-independent endocytosis and trafficking towards Lys via Rab7-positive LE for degradation. In fact, in Rab7-positive LE, ubiquitinylated EGFR is directed into ILVs in order to sequester ligand-bound EGFR away from the limiting membrane and terminate signalling [30].

Hence, the tight coupling of EGFR signalling and trafficking provides multiple opportunities to modulate EGFR signal output in space and time. As Annexins regulate endosomal transport, and can provide the scaffold to create and establish localized signal protein complexes, a substantial amount of studies have described roles for several Annexins in the EGFR/Ras/MAPK signalling pathway. We have previously summarized the numerous protein-protein interactions that are regulated by Annexins that impact on EGFR signalling and trafficking [10,107]. It would go beyond the scope of this review to discuss all of these in detail, but the most prominent examples of Annexins altering EGFR trafficking and signalling are mediated by their scaffolding function, facilitating the recruitment of negative regulators of EGFR and Ras [108,109], or enabling the complex formation of EGFR with phosphatases [35].

At the plasma membrane, the work from our laboratory identified AnxA6 to promote protein kinase Cα-mediated EGFR threonine (T654) phosphorylation, which inhibits EGFR tyrosine phosphorylation and downstream activation of effector pathways [109]. These studies revealed that AnxA6 acts as a scaffold to enable plasma membrane targeting of PKCα and EGFR/PKCα complex formation. AnxA6-dependent EGFR inactivation was associated with reduced EGFR internalization and activation. In addition, we previously demonstrated that AnxA6 also recruits p120GAP, a GTPase activating protein, to the plasma membrane to inhibit Ras signalling downstream of activated EGFR [110]. As AnxA6 is located in EE and LE, one can speculate that AnxA6 might serve as a scaffold to recruit PKCα and p120GAP to endocytic compartments, thus further contributing to downregulate EGFR and Ras activity.

Exemplifying the diversity of how Annexins can modulate EGFR activity, AnxA1 inhibits EGFR signalling through phosphatases, which facilitate EGFR tyrosine dephosphorylation. Protein-tyrosine phosphatase 1B (PTB1B) is one of the phosphatases that can promote EGFR downregulation. However, PTB1B is located in the ER and trafficking of endocytosed EGFR to the ER had not been observed. Yet, ER-MVB contacts were recently identified as sites for PTB1B-mediated EGFR downregulation, preceding sorting of inactive EGFR onto ILVs for degradation [111]. Follow-up studies revealed that ER-MVB contacts were tethered by AnxA1 and its Ca^2+^-dependent ligand, S100A11. AnxA1 is known to associate with EGFR and a well-known substrate for EGFR tyrosine kinase [35,52], indicating that EGFR-mediated AnxA1 phosphorylation might contribute to establish contacts between EGFR-positive endosomes and the ER. Interestingly, the AnxA1-induced microenvironment that enables this EGFR trafficking route is coupled to cholesterol transfer from the ER to MVBs [36] (Figure 1). Moreover, the subsequent step in EGFR downregulation, its removal from the cytoplasm via inward vesiculation in MVBs, requires cholesterol delivery from the ER. While the association of EGFR downregulation with cholesterol transport may only occur when LDL-cholesterol levels in MVBs are low, this clearly highlights the potential of this Annexin to translate nutritional status into the regulation of growth factor receptor activity.

In contrast to the potential of AnxA1 and AnxA6 affecting the localization and activity of EGFR and its effector pathways via direct protein-protein interactions, AnxA2 and AnxA8 probably impact on EGFR within the endocytic pathway indirectly. This includes AnxA2 to modulate cholesterol distribution during EE to LE maturation (see above), and AnxA8 modifying LE morphology and motility [45]. AnxA8 overexpression caused LE/MVB clustering in the perinuclear region, while AnxA8 depletion induced the localization of LE/MVBs in the cell periphery. The latter correlated with impaired EGF-induced EGFR degradation. The underlying cause may involve changes in the actin cytoskeleton [45,46] or LE-cholesterol homeostasis [87], both of which are known to affect LE/MVBs positioning and functioning.

Taken together, several Annexins contribute to fine-tune EGFR activity along the endocytic pathway. Depending on the cell-type or the tissue analysed, their diverse involvement in the regulation of EGFR trafficking, often involving LE-cholesterol, and various scaffolding functions, provide opportunity to differentially regulate EGFR signalling outcomes in growth, differentiation, and many other biological activities.

### 5.3. mTORC1 Signalling from the LE/Lys Compartment

In contrast to the multitude of EGFR signalling events originating from different endocytic sites, other protein complexes only signal from the LE/Lys compartment. This includes the mammalian target of rapamycin complex 1 (mTORC1), which is a critical signalling hub that regulates cell growth and metabolism in response to the availability of nutrients, in particular, amino acids, glucose, and growth factors or the energy status of the cell [112].

Together with mTORC2, mTORC1 enables adaptation to changes in the microenvironment through the upregulation of biosynthetic pathways. This is achieved through mTORC1-mediated phosphorylation of substrates that increase ribosome biogenesis, gene transcription, mRNA translation, carbohydrate and amino acid metabolism, autophagy, as well as microtubule and actin dynamics [113,114]. In addition, mTORC1 activation has significant consequences for lipid metabolism, promoting *de novo* cholesterol and fatty acid synthesis, ensuring membrane biosynthesis for proliferation, and generating lipid stores as energy source for the future synthesis of sterol and fatty acid derivates [115]. Hence, the LE/Lys compartment is not only a sorting station for exogenous cholesterol and other lipids, but mTORC1 activation in LE/Lys also drives anabolic pathways in lipid metabolism.

A great advance in the understanding of mTORC1 activity came from the identification of several Rag GTPases, RagA and B, together with RagC and D, which convert nutrient signals from amino acids and glucose into the recruitment of mTORC1 to Rab7-positive LE/Lys [116,117]. Once at the LE/Lys compartment, the Rheb GTPase then triggers mTORC1 kinase activation [118], followed by the phosphorylation of key regulators that control cell growth.

Although the LE/Lys compartment coordinates sorting of exogenous lipids, the majority of studies in this field have focused on amino acid- and glucose-induced mTORC1 activation, and revealed plenty of consequences for anabolic cholesterol and fatty acid metabolism [115]. Alternatively, it was hypothesized that active mTORC1 in LE/Lys could sense the availability of incoming cholesterol and other lipids through the diet. In support of this model, increased dietary uptake of lipids in mice upregulated mTORC1 activity [119,120]. In addition, LDL uptake is increased in proliferating cells [75,121], which would raise cholesterol content in the LE/Lys compartment. These observations coincide with other studies that demonstrated the changes in the LE/Lys microenvironment, indicating an altered membrane order and function of integrated LE proteins, to influence mTORC1 activity [115,122,123]. Indeed, NPC1 depletion or drug-induced LE-cholesterol accumulation was associated with the inhibition of mTORC1 activity in endothelial cells [124]. In addition, the NPC1-mutant phenotype is associated with defects in endosomal/lysosomal Ca^2+^ homeostasis and thapsigargin, which releases Ca^2+^ from the ER, can correct cholesterol accumulation in NPC1 mutants [125]. This exciting association of Ca^2+^ homeostasis with cholesterol transport in LE/Lys appears to be highly relevant for mTORC1, as thapsigargin restored cholesterol export in LE-cholesterol-rich endothelial cells and reversed the inhibition of mTORC1 signalling [124]. Hence, LE-associated proteins regulated by Ca^2+^ and LE-cholesterol, including Annexins, are attractive candidates that could be responsible for these observations.

Recent studies have further substantiated the ability of mTORC1 to sense LE/Lys-cholesterol levels. In fact, LDL-cholesterol transport to LE/Lys, but not oxysterols or fatty acids, and independent of amino acids, led to the recruitment and activation of mTORC1 [126] (Figure 1). The mode of action involves LE-cholesterol to bind SLC38A9, which is a lysosomal amino acid transporter that is implicated in mTORC1 activation [127,128], translating elevated LE-cholesterol levels into mTORC1 activation. Strikingly, NPC1 interacts with SLC38A9 to control mTORC1 activation and NPC1 depletion resulted in constitutively active mTORC1 activity that could not be stimulated by the addition of LDL [126]. Taken together, these studies strongly indicate that the transport of LDL-derived cholesterol across LE membranes provides a feedback mechanism to control the master growth regulator mTORC1. Although the involvement of Annexins in these settings is yet unknown, we speculate that LE-associated Annexins, in particular, AnxA6 upregulation or AnxA8 depletion, leading to cholesterol accumulation in the LE/Lys compartment, are likely to have an impact on mTORC1 activity.

These observations would provide exciting opportunities to identify novel functions of LE-associated Annexins in cell metabolism and the energy status in health and disease.

## 6. Annexins and Membrane Contact Sites: Close Encounters at the Interface of LE and the ER

In eukaryotic cells, the communication between organelles is fundamental to the cell’s coordinated response to physiological and pathological stimuli. For decades, vesicular membrane trafficking was considered to facilitate the exchange of molecules and information between different organelles. However, this classical view has recently been challenged by the identification of direct physical contacts between organelles as another important and widespread means for cargo exchange [76,77]. MCS are defined as regions of close proximity (10–30 nm) between membranes from two different cellular organelles [129] that allow for the exchange of small molecules, such as lipids and ions. These contact sites do not form randomly, but are transiently established via very specific protein-protein interactions between two organelles. It is generally believed that MCS are built by proteins that are residing in the membrane of organelles. In addition, the recruitment and/or the participation of cytosolic proteins to these domains, which can establish connections or act as tethers, may also contribute to this process. Strikingly, although these interactions can be maintained over time, the fusion between the membranes at these contact sites of different organelles never occurs [130].

MCS exist predominantly between the ER and different endocytic or non-endocytic organelles [111,131,132,133]. In regards to endosomes, MCS contribute to endosome positioning within the cell [134,135,136,137], coordination of endosome motility to control the timing and subcellular location of fission events [138], endosome maturation [139], lipid and Ca^2+^ transfer [77,140,141], and to establish platforms for protein interactions across organelle membranes [36,111,142].

Although NPC1/2 is central to LE-cholesterol egress [67,68], the cholesterol transport routes exiting the LE compartment are still not well defined. This includes the formation of MCS, which transfer cholesterol between LE and the ER [76,77,143,144] (Figure 1). In LE membranes, several proteins are believed to contribute to cholesterol transfer to the ER, including NPC1, oxysterol-binding protein-related protein 1 (ORP1L), StAR-related lipid transfer domain protein 3 (StARD3) and StARD3NL. In addition, the activation of Rab7 (Rab7-GTP) to promote the motility and re-positioning of LE is also critical for MCS formation and cholesterol transfer [76,77]. In the ER, vesicle-associated membrane protein-associated proteins A/B (VAP-A/B), protrudin, and ORP5 are considered as MCS core elements [77,141,145,146]. Current models favour NPC1, together with ORP1L and Rab7, to establish MCS with VAP proteins for LE-ER cholesterol transfer [77,147]. On the other hand, StARD3, together with VAP proteins, also contributes to MCS-mediated cholesterol transfer from the ER to LE [148] (Figure 1).

Despite the greatly improved knowledge on the MCS core elements listed above that enable cholesterol transfer between LE and the ER, there is still a major gap of knowledge, as the feedback loop that allows for dietary LDL-cholesterol uptake to control cholesterol synthesis in the ER would implicate very effective on/off mechanisms that coordinate MCS formation for cholesterol transfer. However, regulatory factors that allow or inhibit core MCS elements to interact for cholesterol transfer are still unknown. 

In this context, the transient and reversible membrane binding behaviour of Annexins could provide a regulatory means to transiently bring LE and ER membranes together. Indeed (see also Section 4), the AnxA1-S100A11 complex can tether subpopulations of endocytic organelles with the ER via ORP1L and VAP-A when the LDL-cholesterol levels in MVBs are low, enabling cholesterol transfer from the ER to MVBs and ensuring ILV formation [36]. It is tempting to speculate that, similar to AnxA1-S100A11-induced LE-ER contact formation, the interaction of other Annexins with members of the S100 protein family [149,150] may also contribute to the establishment of MCS. Given that Annexins form heterotetramers with S100 proteins, which have been proposed to interact with two different membranes simultaneously and allow for membrane fusion [40,150,151], these AnxA-S100 complexes may also have the ability to induce the formation of MCS and allow for the exchange of ions and lipids, including cholesterol.

**Figure 2:**
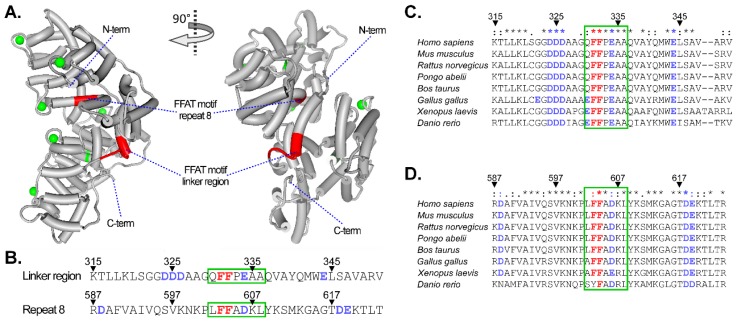
Putative two phenylalanines (FF) in an acidic track (FFAT) motifs in AnxA6; (**A**) Protein structure of bovine AnxA6 (1AVC [5]), showing alpha helixes (tubular structures), Ca^2+^ ions (green) and putative FFAT motifs (red). N- and C-terminus are indicated; (**B**) Amino acid sequence of the FFAT motif-containing regions of AnxA6, highlighting the phenylalanine (FF) residues (red) within the FFAT motif and the negatively-charged amino acids (blue) in the flanking region; ClustalO sequence comparison of (**C**) the AnxA6 linker region and (**D**) repeat eight amino acid sequences of the putative FFAT motifs in different vertebrates. The relative amino acid position is indicated. Symbols represent fully conserved residues (*), conservation between groups of strongly similar properties (:) and conservation between groups of weakly similar properties (.). Green frame highlights putative FFAT motif sequence.

Another possible mechanism how Annexins might contribute to MCS formation emerges from the ER-transmembrane proteins VAP-A and VAP-B. VAP-A/B establish MCS via recognition of FFAT motifs (two phenylalanines (FF) in an acidic track) in proteins residing in endosomes, the Golgi apparatus or peroxisomes. Thereby, the ER can establish contact with LE via ORP1L [133,152,153], StARD3 [154], or protrudin [135]. Interestingly, AnxA6 sequence analysis revealed the presence of two potential FFAT motifs (Figure 2); one FFAT homology (aa 603–604) was found in the C-terminal repeat 8, which is located in an inner zone of AnxA6, with low accessibility, and unless large conformational changes occur, it is unlikely to interact with other proteins. However, the second FFAT homology (aa 331–332) is located in the AnxA6 linker region with opportunities for protein interactions, when considering that this region has been predicted to be away from the plane of the membrane in the presence of Ca^2+^ [5,6]. It should be noted that the algorithm that was designed to identify FFAT motifs [155] only indicated a weak potential of the FF sequence within the AnxA6 linker region to interact with VAP-A (Dr. Tim Levine, personal communication). However, this is not uncommon, as other proteins that are known to interact with VAP-A/B, such as StARD2, ORP10, and ORP11, are also characterized by weak scores when applying the abovementioned algorithm.

In addition, the main discrepancy between the published FFAT consensus [155] and the AnxA6 FFAT motif in the linker region include (i) the three spacer residues AAG separating the acidic (DDD) and FF residues, and (ii) a P residue separating FF from the acidic EAAQ sequence (Figure 2). The AAG spacer with its small side chains may provide sufficient flexibility for the acidic residues, which could then still interact with the FF pair as in a normal FFAT motif. Likewise, the P residue separating FF and EA causes a turn that might similarly permit the approximation of the FF and EA residues (Reginald Morgan, personal communication). Thus, future studies should address the potential of the FFAT motifs in AnxA6 for their ability to support membrane contact formation between LE and the ER, possibly via interaction with ER-resident proteins, or via NPC1 [79,156], which interacts with ORP5 to form MCS structures for cholesterol transfer between the LE and ER [157,158,159].

## 7. Annexins and Biogenesis of Exosomes

The LE compartment is not only central to the cellular distribution of cholesterol, and the delivery of membrane and cargo to Lys along the endocytic pathway, but also in the generation of exosomes, nanovesicles that are secreted by cells, and are increasingly recognized as new mediators for intercellular communication. Exosome biogenesis occurs in LE/MVBs, and their precursors (ILVs) are generated by inward budding. This is followed by the trafficking of MVBs to the plasma membrane, and finally, the secretion of ILVs, then becoming exosomes [160]. For further reading, we refer to excellent reviews on exosome biogenesis [161]. Below, we will highlight links that connect LE-associated Annexins with the biogenesis and secretion of exosomes.

Interestingly, exosomes do not only carry proteins, RNA, and lipid second messengers, but also cholesterol [162]. In fact, exosomes are known for their high cholesterol content and can contribute to cholesterol accumulation that can modify lipid homeostasis in recipient cells [163]. Importantly, cholesterol is required for several steps in the biogenesis of exosomes. This is a complex process, because ILV formation needs to be coordinated with the loading of ILV with a variety of molecules, including signalling proteins, nucleic acids, or cytoplasmic material [161]. Adding another level of complexity, subsets of ILVs coexist even within a single MVB, with each being characterized by different lipids and size. This is probably due to a diversity in the mechanisms that can trigger ILV formation [164,165,166], which is still not well understood. The sequential action of different components of the endosomal-sorting complexes that are required for transport (ESCRT), together with ubiquitination, is the best-described machinery that drives ILV formation [167,168]. In addition, tetraspanins (CD63, CD81, CD82) [169,170,171], ceramide [172], and lysobisphosphatidic acid (LBPA) [173] contribute to different MVB subpopulations that are destined for either lysosomal degradation or exosomal release [174].

Several of the mechanisms that are listed above are interrelated to cholesterol. ILVs accumulate cholesterol [175,176], and ESCRT complexes generate cholesterol-rich microdomains [177] that contribute to the trafficking of the tetraspanin CD82. The LBPA-interacting protein Alix controls the MVB cholesterol content [178], and most intriguingly, in certain cell types, drug (U18666A)- or NPC1-induced LE-cholesterol accumulation favours cholesterol secretion via exosomes [179]. Hence, exosome secretion can bypass cholesterol accumulation, possibly contributing to maintain cholesterol homeostasis. 

In addition, and related to AnxA6-induced LE-cholesterol accumulation and its functional consequences for cholesterol-dependent cellular events at the plasma membrane [79,80], a recent study deciphered the mechanisms that contribute to exosome secretion [180]. MVBs are equipped with specific SNARE proteins that enable fusion with the plasma membrane to stimulate exosome release. At the plasma membrane, this fusion process requires syntaxin 4 and SNAP23, which correlates with findings from our laboratories, demonstrating the mislocalization and dysfunction of these two SNARE proteins upon AnxA6-mediated alteration of cellular cholesterol distribution [80].

Taken together, and although a distinct role for Annexins in the formation/assembly or the secretion of exosomes has yet to be demonstrated, the latter findings strongly associate the impact of LE-associated Annexins AnxA6 and AnxA8 on LE-cholesterol export [79,87] with exosome biogenesis. Also, the contribution of AnxA1 and AnxA2 in the regulation of trafficking of MVB subpopulations [36,52,56,58,111] is likely to determine ILV destiny along degradative or secretory routes.

Besides cholesterol, exosomes contain substantial amounts of negatively charged phospholipids, including PS, PI, and PA [162]. This correlates with the proteomic analysis of isolated exosomes, identifying a repertoire of phospholipid-binding Annexins, including AnxA1, A2, A4, A5, A6, and A11, being among the 100 most abundant proteins found in exosomes [181]. In certain situations, such as LE enlargement due to cholesterol accumulation, some Annexins have also been observed inside LE/Lys, for example, AnxA2 [182] and AnxA6 [183,184], enabling the putative location of these Annexins in the outer leaflet of exosomes.

Interestingly, increasing numbers of reports have identified Annexins to associate with RNAs. This interaction is particularly relevant for miRNA loading of exosomes and may contribute to the targeting of Annexins to the lumen of exosomes. Up to date, evidence exists for AnxA2 to influence sequence-independent loading of miRNAs into extracellular vesicles [185]. These features are probably not related to the Ca^2+^- or cholesterol-dependent membrane association that is discussed above, yet greatly extend the diversity of Annexin-related functions. Thus, the ability of AnxA2 and possibly other LE-associated Annexins to recruit miRNAs as well as other proteins into exosomes implicates them in control of cell-cell communication in health and disease [186].

## 8. Annexins and LE/Lys Positioning 

It is now well believed that LE maturation and LE/Lys function is highly dependent on the distribution of LE/Lys within cells. LE motility determines the position of LE/Lys, and it reflects the response to a variety of stimuli. Markedly, alterations in this regulation seem to be associated with different pathologies related to cell adhesion and motility, as well as autophagy [187,188]. Thus, an evolving theme of interest in the field is how LE/Lys function may be influenced by LE/Lys positioning. 

LE/Lys move bi-directionally on the microtubule network by dynein and kinesin motors. The microtubule-organizing center is the minus-end, and in general, in non-polarized cells, is located between the Golgi and the nucleus, whereas the microtubule plus-ends are located at the cell periphery (plasma membrane). Therefore, minus-end directed microtubule motors, such as dynein, move LE/Lys from the periphery to the cell centre, while the plus-end directed microtubule motors, kinesins, promote the scattering of LE/Lys throughout the cytoplasm. These opposing forces determining LE/Lys movements are connected to the energy and dietary status of cells, as starvation promotes autophagosomes and LE/Lys to move towards the cell centre for fusion and subsequent degradation of the autophagosomal content [189,190]. On the other hand, the redistribution of LE/Lys towards the cell periphery appears vital for growth, migratory, and invasive properties of cells, ensuring the rapid delivery of membrane and secretory components to the cell surface. Indeed, plus-end (anterograde) transport facilitates lysosomal exocytosis, leading to the secretion of acidic hydrolases and metalloproteinases that degrade the extracellular matrix to promote the migration and invasion of cancer cells [191,192,193].

Several protein complexes have been implicated in the regulation of LE/Lys positioning, and have been described in detail [194,195]. Most relevant here, this also includes Rab7, together with different effectors to either promote plus-end or minus-end (retrograde) directed LE/Lys movement. Active Rab7, together with PIP2 and the FYVE and coiled-coil domain containing 1 (FYCO1) adaptor protein in LE, binds the ER-resident protein protrudin. This complex recruits kinesin-1, which promotes plus-end trafficking [76,77,135,196]. On the other hand, Rab7 and its effector Rab-interacting lysosomal protein (RILP) enable retrograde movement. RILP interacts with the p150-glued subunit of dynactin, which then recruits the minus-end directed microtubule motor dynein to LE/Lys [134,197,198].

These opposing Rab7 activities must be coordinated, and reflect the yet limited knowledge on factors that modulate the diversity of Rab7 functions [199,200]. The factors that determine Rab7 to interact with either kinesin or dynein motor proteins to influence LE motility and LE/Lys positioning are still unknown. However, insights from LE-cholesterol homeostasis provide striking mechanistic details, as LE move to microtubule plus-ends (cell periphery) when LE-cholesterol levels are low, whereas high LE-cholesterol levels promote LE to move towards the minus-end (cell centre) [76,77]. Hence, in cholesterol-rich LE of NPC1 mutant cells, Rab7 together with the dynein machinery may ensure positioning of large, cholesterol-rich LE/Lys at the cell centre. Under these conditions, low Rab7-GTP levels would not allow formation of Rab7-GTP/PIP2/FYCO1 complexes and interaction with protrudin, thereby blocking recruitment of kinesin-1 for endosome trafficking. On the other hand, low LE-cholesterol levels correlate with elevated Rab7-GTP, reduced LE size, elevated LE/Lys motility, indicating Rab7, protrudin, and kinesin-1 dependent LE/Lys trafficking to the periphery. Thus, LE-cholesterol accumulation induced by up- or downregulation of AnxA6 and AnxA8, respectively [79,87], and AnxA6-mediated downregulation of Rab7-GTP levels, could be decisive factors that determine the ability of Rab7 to influence LE/Lys positioning. 

Extending these observations further, and given that Rab7 is pivotal for the cholesterol-dependent establishment of MCS (see Section 6), one can envisage that MCS formation between LE/Lys and the extensive ER network [130,201] could contribute to LE/Lys repositioning.

Although the scenarios that are described above provide attractive models of how LE-associated Annexins might affect the distribution of LE/Lys in cells, the picture is still incomplete and several other players and pathways need to be considered. This also includes a possible indirect role of the actin cytoskeleton influencing cargo transport from EEs to LEs [31], sorting and vesicle fission in the recycling pathway, and cargo transport to the trans-Golgi-network and lysosomes [202,203,204]. We and others have extensively reviewed how Annexins interact and re-arrange membrane-actin interactions [13,40], in particular, at endosomal membranes [45,58]. Possibly complexed with S100 proteins, these links to the cytoskeleton may thus provide tethering between vesicles and/or contribute to the repositioning of LE/Lys.

Finally, the possibility that LE-associated Annexins may influence integral LE/Lys proteins that could provide coupling to motor proteins for retrograde lysosomal transport should also be mentioned. Interestingly, this includes proteins that are associated with cholesterol export from LE/Lys, such as lysosomal-associated membrane protein 1 and 2 [205,206] and many others, [207,208,209], but also Ca^2+^ channels and Ca^2+^-binding proteins [210]. Along these lines, several Annexins interact with the proton pump H^+^-ATPase subunit VOa2 [211] and the TPC1/2 Ca^2+^ channels [66]. These latter interactions might influence how Ca^2+^ is provided for membrane fusion between LE/Lys and other compartments, including the plasma membrane. In this context, Ca^2+^ sensors, including Annexins, may allow for lysosomal Ca^2+^ release to regulate the distinct steps of lysosomal trafficking [212]. As cholesterol accumulation in NPC1-mutant cells blocks endosomal/lysosomal Ca^2+^ release [125], these Ca^2+^-regulatory circuits to alter LE/Lys positioning are likely to be connected to LE-cholesterol levels.

## 9. Concluding Remarks and Future Perspectives

In this review, we have summarized some of the current knowledge that implicates Annexins in a variety of processes in the LE/Lys compartment that are linked to LE-cholesterol transport and the impact on endosomal membrane traffic, endosome maturation, signal transduction, cholesterol homeostasis, tethering and MCS formation, and LE/Lys positioning. Our knowledge is still incomplete, but a subset of Annexins seems to converge and sense Ca^2+^ and cholesterol alterations in LE/Lys to perform a variety of cellular functions. AnxA1 is required to tether endocytic vesicles with the ER, which allows transfer of cholesterol to MVBs, ensuring ILV formation. AnxA2-dependent and cholesterol-driven endosome maturation ascertains the onset of degradation. Increased AnxA6 expression and recruitment to LE membranes induces LE-cholesterol accumulation. On the other hand, AnxA8 depletion blocks LE-cholesterol egress. The molecular mechanisms that are involved in this regulation is closer to being clarified. As discussed, these diverse modes of action mediated by the various Annexins and affecting LE-cholesterol homeostasis have multiple consequences for a variety of cellular activities. Given the involvement of Annexins in all of these regulatory circuits that modulate LE/Lys function, including the possibility of gene defects, Annexins could contribute to disease-related phenotypes that are observed in LSD, neurodegeneration, or cancer.
